# Predictive Value of the *TP53*/*PIK3CA*/*ATM* Mutation Classifier for Patients With Bladder Cancer Responding to Immune Checkpoint Inhibitor Therapy

**DOI:** 10.3389/fimmu.2021.643282

**Published:** 2021-08-04

**Authors:** Yi-Hui Pan, Jia-Xing Zhang, Xu Chen, Fei Liu, Jia-Zheng Cao, Yu Chen, Wei Chen, Jun-Hang Luo

**Affiliations:** ^1^Department of Urology, First Affiliated Hospital, Sun Yat-sen University, Guangzhou, China; ^2^Department of Oncology, First Affiliated Hospital, Sun Yat-sen University, Guangzhou, China; ^3^Department of Urology, National Cancer Center/National Clinical Research Center for Cancer/Cancer Hospital, Chinese Academy of Medical Sciences and Peking Union Medical College, Beijing, China; ^4^Department of Urology, Jiangmen Hospital, Sun Yat-sen University, Jiangmen, China

**Keywords:** bladder cancer, immune checkpoint inhibitor, mutation profile, immunotherapy, immune cell infiltration, signature

## Abstract

**Background:**

Only a proportion of patients with bladder cancer may benefit from durable response to immune checkpoint inhibitor (ICI) therapy. More precise indicators of response to immunotherapy are warranted. Our study aimed to construct a more precise classifier for predicting the benefit of immune checkpoint inhibitor therapy.

**Methods:**

This multi-cohort study examined the top 20 frequently mutated genes in five cohorts of patients with bladder cancer and developed the *TP53*/*PIK3CA*/*ATM* mutation classifier based on the MSKCC ICI cohort. The classifier was then validated in a validation set consisting of IMvigor210 cohort and Broad/Dana-Farber cohort. The molecular profile and immune infiltration characteristics in each subgroup as defined by this classifier were explored.

**Results:**

Among all 881 patients with bladder cancer, the mutation frequency of *TP53*, *PIK3CA*, and *ATM* ranked in the top 20 mutated genes. The *TP53*/*PIK3CA*/*ATM* mutation classifier was constructed based on the Memorial Sloan Kettering Cancer Center (MSKCC) ICI cohort and only showed predictive value for patients with bladder cancer who received ICI therapy (median overall survival: low-risk group, not reached; moderate-risk group, 13.0 months; high-risk group, 8.0 months; P<0.0001). Similar results were found in subgroups of MSKCC ICI cohort defined by tumor mutation burden. Multivariate Cox analysis revealed that the risk group defined by the classifier served as an independent prognostic factor for overall survival in patients with bladder cancer. Efficacy of the classifier was verified in a validation set consisting of IMvigor210 cohort and Broad/Dana-Farber cohort. Lower expression of PD-1/PD-L1 and less tumor immune infiltration were observed in the high-risk group than the other two groups of the TCGA cohort and the IMvigor210 cohort.

**Conclusion:**

Our study constructed a *TP53*/*PIK3CA*/*ATM* mutation classifier to predict the benefit of immune checkpoint inhibitor therapy for patients with bladder cancer. This classifier can potentially complement the tumor mutation burden and guide clinical ICI treatment decisions according to distinct risk levels.

## Introduction

Recently, an increasing number of researchers focused their attention on the relationship between tumor progression and the immune status. Immune checkpoint inhibitors (ICIs) have become the most promising therapeutic modality for patients with malignant neoplasms, including bladder cancer (BC). Antibodies targeting programmed cell death 1 (*PD-1*) (OMIM 600244)/programmed death-ligand 1 (*PD-L1*) (OMIM 6005402) and cytotoxic T-lymphocyte associated protein 4 (*CTLA-4*) (OMIM 123890) have shown high therapeutic efficacy. Over the past few years, five new ICIs have been approved for the second-line systemic therapy in the locally advanced or metastatic BC diseases (1). Unfortunately, only a proportion of unselected BC patients showed obvious improvement (2–5). In a phase 2 multicenter study, the objective response rate to atezolizumab (a PD-L1 inhibitor) was 15%, regardless of the expression of PD-L1 (3). Similar results were observed in the CheckMate 275. In this phase 2 trial of nivolumab (a PD-1 inhibitor), the objective response was confirmed as 19.6% (4). In an international phase 3 trial of pembrolizumab (a PD-1 inhibitor), the objective response rate was 21.1% (5). Therefore, researchers started to focus on finding new biomarkers for treatment stratification. Encouragingly, recent studies have identified some positive predictive biomarkers for ICI therapy, such as tumor mutation burden (TMB) and microsatellite instability (MSI) (6–8). High levels of TMB and MSI may be associated with accumulation of neoantigen and stimulate the immune response, resulting in favorable response to ICI therapy. Gene mutation signatures have also been gradually identified as a good complement (9, 10).

Nevertheless, few verified biomarkers of the response to ICI therapy in BCs have been reported. In the Checkmate 275 study, higher values of the 25-gene interferon-γ (IFN-γ) signature were associated with higher PD-L1 expression and improved response rate to nivolumab (4). Min et al. elucidated an association between the alterations in DNA damage response and repair (DDR) genes and response to ICI in advanced urothelial carcinomas (UC), whereas *ATM* was the most commonly altered genes among the DDR-related genes (11). Thiago et al. also found that, in muscle invasive bladder cancers, mutations of DDR genes were associated with the expression of tumor immune regulatory gene expression (12). Besides, Sangeeta et al. reported *ARID1A* mutation plus *CXCL13* expression as composite biomarkers to predict responses to ICI therapy in metastatic UC (13). Composite somatic mutations seem to be potential biomarkers in advanced or metastatic BCs. However, these observations still need to be validated in a larger cohort for future development.

Herein, we aimed to screen the most commonly mutated genes in BC patients and constructed a novel gene mutation classifier to predict the benefit of immune checkpoint inhibitor therapy more precisely. The mutation classifier was validated in an independent validation set. Comprehensive bioinformatics analyses were carried out to understand the underlying mechanisms and potential prognostic value of the classifier.

## Materials and Methods

### Patients and Samples

Somatic mutation data and clinical data of patients with BC from the Memorial Sloan Kettering Cancer Center (MSKCC) ICI cohort (n=215) (6)^1^, MSKCC non-ICI cohort (n=172) (14)^2^, The Cancer Genome Atlas (TCGA) cohort (n=412) (15)^3^, ^4^, Weill Cornell Medicine/University of Trento (Cornell/Trento) cohort (n=32) (16)^5^, Dana-Farber Cancer Institute/MSKCC (DFCI/MSKCC) cohort (n=50) (17)^6^, and Broad/Dana-Farber cohort(n=26) (18)^7^ were downloaded from the cBioPortal and the TCGA data portal (19). Patients from the MSKCC center were distinguished by patient ID, history of drug use, and other clinical characteristics for fear of overlapping cases. Gene expression data in fragments per kilobase of transcripts per million mapped reads (FPKM) for 408 samples in the TCGA database were also obtained from the cBioPortal. Somatic mutation data, RNA-seq data, and matched clinical data of BC patients from IMvigor210 cohort (n=237) were obtained from IMvigor210CoreBiologies, a fully documented R package (20)^8^. 215 patients from the MSKCC ICI cohort were assigned to the training set. 263 patients from the IMvigor210 cohort and Broad/Dana-Farber cohort were assigned to the validation set. All the patients from the MSKCC ICI cohort, IMvigor210 cohort, and Broad/Dana-Farber cohort received at least one dose of ICI therapy. Patients from the training set and the validation set with incomplete survival information, mutation data, and TMB data were excluded. The study was conducted according to the Strengthening the Reporting of Observational Studies in Epidemiology (STROBE) reporting guideline.

### Construction of the Mutation Classifier

First, univariate Cox regression analysis was conducted in the top 20 most commonly mutated genes of 881 BC patients from five cohorts. Then, genes with a *P* value less than 0.05, which was determined by univariate Cox regression, were screened to perform a multivariate cox regression analysis. The risk score was calculated with the formula below:

Risk score = (beta_1_× mutation status of Gene_1_) + (beta_2_× mutation status of Gene_2_) + … + (beta_n_× mutation status of Gene_n_).

A mutated gene was coded as 1, and a wild type gene was coded as 0. Beta was the regression coefficient generated in the multivariate Cox regression analysis.

### Division of Risk Scores With the X-Tile Software

X-tile software version 3.6.1 (Camp/Rimm, Yale University) described the substantial tumor subpopulations *via* dividing a population into three risk score levels (low-, moderate-, and high-level) (21). X-tile plot was shown in a right triangular grid, where each pixel represented a different cutoff point. Each division had a Chi-Sq (χ^2^) value, which was shown with a color code on the grid. The X-tile software could automatically select the optimal division through χ^2^ value. A *P* value calculated by standard Monte Carlo simulations was used to assess statistical significance.

### Assessment of TMB

All non-synonymous mutations, including missense, frame-shift, nonsense, nonstop, splice site, and translation start site changes of *TP53*/*PIK3CA*/*ATM*, were considered. TMB was defined as the total number of somatic non-synonymous mutations normalized to the total number of megabases sequenced.

We collected TMB data of patients from the MSKCC ICI cohort, which generated from the Memorial Sloan Kettering-Integrated Mutation Profiling of Actionable Cancer Targets (MSK-IMPACT). A total of 215 patients with BC, whose tumors were profiled by next-generation sequencing. Genomic alterations of patients from the IMvigor210 cohort were assessed by FMOne panel (Foundation Medicine, Inc.). TMB data of patients from the Broad/Dana-Farber cohort were determined as the total number of mutations per sample, normalized by whole-exome sequencing (WES) coverage.

### Oncoplot of the Mutated Genes

Mutation Annotation Format (MAF) files of BC patients were downloaded from the cBioPortal.

The oncoplot and summarized information were then graphed through the Maftools package in the R version 4.0.4 (22).

### Construction of the Protein–Protein Interaction (PPI) Network

The PPI network functional enrichment analysis was conducted on the STRING website^9^ and reconstructed using the Cytoscape software version 3.8.0 (23).

### Gene Set Enrichment Analysis (GSEA)

The GSEA software version 4.1.0 (Broad Institute, Cambridge, MA, USA) was used to identify the notably altered gene sets between the pre-defined low-risk group and high-risk group in the TCGA cohort. Hallmark gene sets(hallmark gene sets as Gene Symbols), C2: curated gene sets (KEGG gene sets as Gene Symbols) and C7: immunologic signatures(ImmuneSigDB gene sets as Gene Symbols), which represented cell states and perturbations within the immune system, were applied to investigate the alteration in immune-related pathways. Gene expression profiles of the TCGA cohort with grouping information were prepared for GSEA. A *P* value < 0.05 and a false discovery rate (FDR) <0.25 were considered statistically significant.

### Assessment of Immune Infiltration

The ESTIMATE algorithm was applied to calculate the stromal scores, immune score, estimate scores, and tumor purity, which depicted the fraction of stromal and immune cells in tumor samples using expression signatures (24).

The CIBERSORT algorithm was applied to characterize the immune cell composition of complex tissues via an LM22 gene signature matrix (25). The matrix contains 547 genes that distinguish 22 human hematopoietic cell phenotypes. The standardized processed gene expression profiles from TCGA database and IMvigor210CoreBiologies R package were uploaded to the CIBERSORT website^10^ as mixture files. The LM22 signature matrix file was used to run CIBERSORTx, with 1000 permutations. Quantile normalization was disabled on RNA-seq data. The results of CIBERSORT included subsets of seven T cell types, naive and memory B cells, plasma cells, natural killer cells, and myeloid subsets. CIBERSORT conducted deconvolution with Monte Carlo sampling and derived an empirical *P* value. All 408 TCGA samples with gene expression data had *P* values < 0.05. In IMvigor210 cohort, 153 samples had *P* values < 0.05, and 84 samples had *P* values ≥ 0.05. Only samples with a *P* value less than 0.05 were included for the following analyses.

The TIMER algorithm was applied to estimate immune infiltrations (26, 27). The standardized processed gene expression profiles were uploaded to the TIMER2.0 website^11^. The results contained subsets of B cells, CD4+ T cells, CD8+ T cells, neutrophils, macrophages, and myeloid dendritic cells.

### Statistical Analysis

Overall survival (OS) was measured from the date of ICI therapy initiation to the time of death or latest follow-up. For survival analysis, Kaplan–Meier survival curves were generated and compared using the log-rank test. Cox regression analysis was used to establish a multi-gene mutation classifier. Overall survival and overall patient survival status were used as the dependent variables in the univariate and multivariate cox regression analyses. X-tile plots were used to automatically select the optimal division of the classifier by selecting the highest χ^2^ value. The cutoff point of TMB was defined by the quintile (top 20%). The two-tailed unpaired t-test and Kruskal–Wallis test were used to determine the differences between different groups with or without normal distribution, respectively. The Pearson χ^2^ test was applied to estimate the correlations among various immune cell subsets. The SPSS software version 25.0 (IBM Corp., Armonk, NY, USA) and GraphPad Prism version 8.3.0 (GraphPad Software Inc., San Diego, CA, USA) were used to carry out the statistical analysis. All the statistical outcomes were two-sided, with *P* values < 0.05 denoting statistically significant differences. Data were collected and analyzed from March 14, 2020 to June 21, 2021.

## Results

### Distribution and Clinical Significance of BC Gene Mutation Profile Landscape

**Figure 1:**
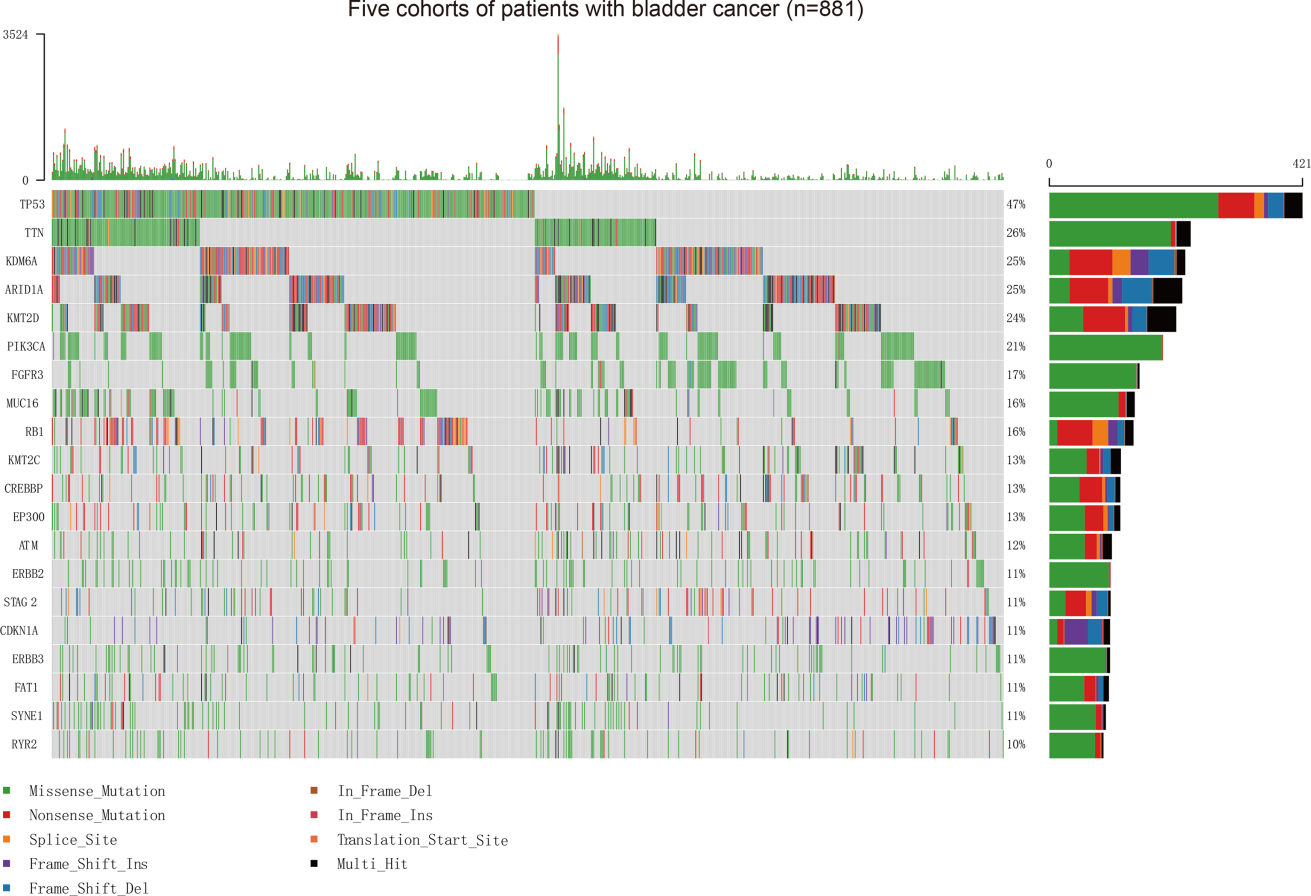
Assessment of the Frequency of Mutated Genes and Mutation Patterns in Patients with BC from Five Cohorts. Oncoplot for the mutated genes of 881 patients with BC from five cohorts. The top 20 genes are listed by mutation frequencies. BC, bladder cancer.

Our study firstly collected somatic mutation data of 881 patients with BC, including 215 patients from the MSKCC ICI therapy cohort (mean [SD] age, 67.6 [9.9] years; 164 [76.3%] men), 172 patients from the MSKCC non-ICI therapy cohort (123 [71.5%] men), 412 patients from the TCGA cohort (mean [SD] age, 68.1 [10.6] years; 412 [73.5%] men), 32 patients from the Cornell/Trento cohort (mean [SD] age, 68.3 [9.4] years; 24 [75.0%] men), and 50 patients from the DFCI/MSKCC cohort (mean [SD] age, 62.5 [8.9] years; 37 [74%] men) (**Supplementary Figure 1**). The vast majority of variant classifications consisted of missense and nonsense mutations. Single nucleotide polymorphism is the most common variant type in BC (**Supplementary Figure 2**). The oncoplot showed the top 20 frequently mutated genes in these five BC patient cohorts (**Figure 1**). To better understand the interplay between frequently mutated genes, we constructed PPI networks of the top 20 mutated genes *via* the STRING database and subsequently conducted an analysis using Cytoscape (**Supplementary Figure 3**). Tumor protein p53 (*TP53*) (OMIM 191170), phosphatidylinositol-4,5-Bisphosphate 3-Kinase Catalytic Subunit Alpha (*PIK3CA*) (OMIM 171834), AT-Rich Interaction Domain 1A (*ARID1A*) (OMIM 603024), ataxia-telangiectasia mutated (ATM) (OMIM 607585), and Lysine Methyltransferase 2D (*KMT2D*) (OMIM 602113) were identified to possess higher stress and more edgecounts than other genes, which implied their central position and complex interactions in the PPI network of BC.

### Predictive Value of the *TP53/PIK3CA/ATM* Mutation Classifier in Patients With BC Receiving ICI Therapy

To investigate the gene signatures of patients sensitive to ICI therapy, we selected the MSKCC ICI therapy cohort as the training set, where each patient underwent PD-1/PD-L1 inhibitor therapy or combination treatment with a CTLA-4 inhibitor. Somatic TMB, measured using the MSK-IMPACT, was provided in the clinical patient information. We firstly utilized univariate cox regression analyses to examine the top 20 mutated genes. *TP53*, *PIK3CA*, *ATM*, and *CREBBP* showed statistical significance (**Supplementary Table 1**). After multivariate adjustment to 4 potential prognostic factors, only *TP53*, *PIK3CA*, and *ATM* showing independent predictive value (*P*<0.05) were selected as prognostic candidates (**Supplementary Table 2**). *CREBBP* had a *P* value > 0.05 and was excluded. Finally, *TP53*, *PIK3CA*, and *ATM* were included in the multivariate survival analysis, and we could generate a risk score for each patient with the cox regression coefficients in the model (**Supplementary Table 3**):

Risk Score=(−0.492*TP53)+ (0.562*PIK3CA)-(1.454*ATM)

The risk scores of the 215 patients in the training set ranged from –1.946 to 0.562.

**Figure 2:**
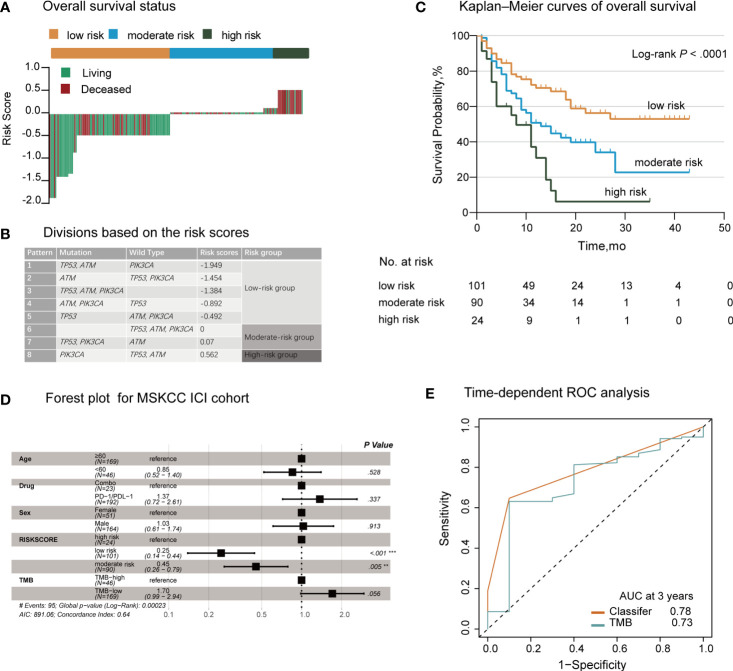
Establishment of the *TP53/PIK3CA/ATM* Mutation Classifier in BC Patients Treated with Immune Checkpoint Inhibitors. **(A)** OS status in the MSKCC ICI cohort. “Living” and “Deceased” patients were marked green and red, respectively. **(B)** Schematic diagram of the divisions based on risk scores. **(C)** Kaplan–Meier curves of overall survival in the MSKCC ICI cohort based on the *TP53/PIK3CA/ATM* mutation classifier. The median overall survival was 8.0 months (95% CI: 13.1–20.9 months) in the high-risk group, 13.0 months (95% CI: 7.8–18.2 months) in the moderate-risk group, and not reached in the low-risk group. **(D)** Forest plot for 215 patients who received ICI therapy. The vertical line represents the hazard ratio (HR) of 1.0. **(E)** Time-dependent ROC curves and AUCs at 3 years were used to assess the predictive accuracy of the classifier compared with TMB. AUC, area under the curve; BC, bladder cancer; CI, confidence interval; ICI, immune checkpoint inhibitors; MSKCC, Memorial Sloan Kettering Cancer Center; OS, overall survival; ROC, receiver operating characteristic; TMB, tumor mutation burden.

We evaluated the distribution of the risk score for the *TP53/PIK3CA/ATM* mutation classifier and survival status in patients who received ICI therapy. Patients with lower risk scores generally showed better response to ICI therapy than those with higher risk scores (**Figure 2A**). We next used the X-tile plots to determine the optimal cutoff point (**Supplementary Figure 4**). Patients with a score >0.07 or <0 were allocated to the high- and low-risk group, respectively. The remaining patients were allocated to the moderate-risk group (**Figure 2B**). Compared with patients in the moderate- and high-risk groups, those in the low-risk group exhibited better therapeutic response and OS (median OS, low-risk group: not reached; moderate-risk group: 13.0 months; high-risk group: 8.0 months; *P*<0.0001) (**Figure 2C**). According to the forest plot, we could confidently conclude that the risk group was a strong indicator of favorable OS among patients with BC (hazard ratio: 1.79; 95% CI: 1.37–2.34; *P*<0.0001) (**Figure 2D**). After multivariable adjustment, the risk group remained an independent predictive factor (hazard ratio: 1.78; 95% CI: 1.35–2.36; *P*<0.0001) (**Supplementary Table 4**). When assessing the predictive accuracy of the classifier, the area under the curve (AUC) of the classifier and TMB at 3 years was 0.78 (95% CI: 0.69–0.88) and 0.73 (95% CI: 0.56–0.89), respectively (**Figure 2E**). Pearson correlation analysis showed a negative correlation between the risk score and TMB (r=−0.25; *P*=0.0002) (**Supplementary Figure 5**).

### Subgroup Analysis of the *TP53*/*PIK3CA*/*ATM* Mutation Classifier in the MSKCC ICI Cohort by TMB

**Figure 3:**
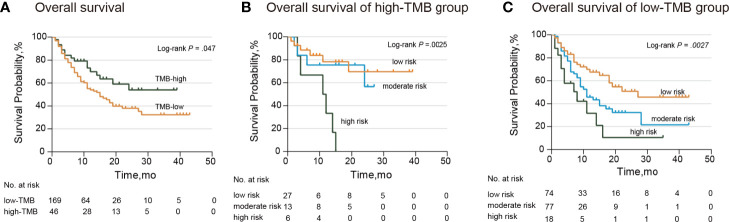
Subgroup Analysis of Overall Survival in the MSKCC ICI cohort stratified by TMB. **(A)** Kaplan–Meier curves of overall survival in patients with BC by TMB. The median overall survival was 15.0 months (95% CI: 10.6–19.4 months) in the low-TMB group and not reached in the high-TMB group. **(B)** Kaplan–Meier curves of overall survival in patients from the high-TMB group. The median overall survival was 11.5 months (95% CI: 1.4–20.6 months) and not reached in the other two groups. **(C)** Kaplan–Meier curves of overall survival in patients from the low-TMB group. The median overall survival was 27.0 months (95% CI: not available) in the low-risk group, 11.0 months (95% CI: 7.5–14.5 months) in the moderate-risk group, and 7.0 months (95% CI: 10.6–19.4 months) in the high-risk group. BC, bladder cancer; CI, confidence interval; TMB, tumor mutation burden.

The median and range of TMB varied across tumor types (28). For this reason, we selected the higher TMB quintile (top 20%) as the cutoff point. According to the TMB cutoff (17.6), we divided patients into the high- and low-TMB groups. Patients in the low-TMB group showed notably poorer response and survival (median OS: low-TMB group, 15.0 months; high-TMB group, not reached; hazard ratio: 1.62; 95% CI: 1.00–2.60; *P*=0.047) (**Figure 3A**). When stratified by the TMB status, our *TP53*/*PIK3CA*/*ATM* mutation classifier became a more precise model for identifying patients sensitive to ICI therapy. In the high-TMB group, we observed that patients in the high-risk group showed the worst response to ICI therapy (median OS, low-risk group: not reached; moderate-risk group: not reached; high-risk group: 11.5 months; *P*=0.0025) (**Figure 3B**). Correspondingly, in the low-TMB group, patients in the low-risk group showed the best response to ICI therapy (median OS, low-risk group: 27.0 months; moderate-risk group: 11.0 months; high-risk group: 8.0 months; *P*=0.0027) (**Figure 3C**).

After adjusting for sex, age, and ICI treatment through multivariate Cox regression analysis, the classifier remained an independent prognostic factor in both subsets (**Supplementary Table 5**).

### Validation of the *TP53*/*PIK3CA*/*ATM* Mutation Classifier

**Figure 4:**
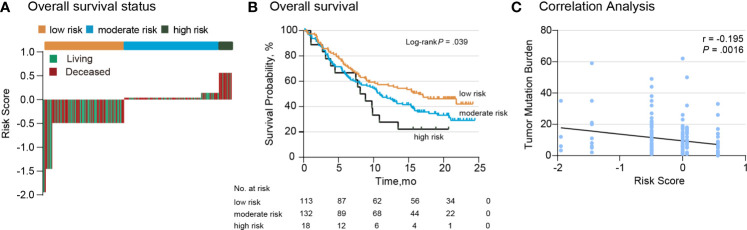
Validation of the *TP53/PIK3CA/ATM* Mutation Classifier. **(A)** OS status in the validation set. “Living” and “Deceased” patients were marked green and red, respectively. **(B)** Kaplan–Meier curves of overall survival in the validation set based on the TP53/PIK3CA/ATM mutation classifier. The median overall survival was 8.1 months (95% CI: 5.8–10.4 months) in the high-risk group, 10.9 months (95% CI: 8.2–13.6 months) in the moderate-risk group, and 16.5 months (95% CI: 10.1–22.8 months) in the low-risk group. **(C)** Correlation between the risk score and TMB in the validation set.

To estimate the efficacy of the *TP53*/*PIK3CA*/*ATM* mutation classifier, we tested the classifier in a 263-cases validation set. The validation set consisted of 237 BC patients from the IMvigor210 cohort (191 [80.6%] men) and 26 BC patients from the Broad/Dana-Farber cohort (18 [69.2%] men). All the patients in the validation set underwent ICI therapy. We once again evaluated the distribution of the risk score for the *TP53/PIK3CA/ATM* mutation classifier and survival status in the validation set. Patients with lower risk scores benefited more from the ICI therapy (**Figure 4A**), in accordance with the results of the training set. Patients in lower risk group showed better therapeutic response and OS (median OS, low-risk group: 16.5 months; moderate-risk group: 10.9 months; high-risk group: 8.1 months; *P* =0.039) (**Figure 4B**). A negative correlation between risk scores and TMB was also found in the validation set (**Figure 4C**). Univariate and multivariate cox regression analyses of BC patients in the validation set confirmed the results as well (**Supplementary Table 6**).

### *TP53*/*PIK3CA*/*ATM* Mutation Classifier in Patients From the Non-ICI Therapy Cohort

When extended to patients with BC from the MSKCC non-ICI cohort, the *TP53*/*PIK3CA*/*ATM* mutation classifier did not show significant differences between the three groups (median OS, low-risk group: not reached; moderate-risk group: 30.5 months; high-risk group: not reached; *P*= 0.27) (**Supplementary Figure 6A**). Similar results were noted in patients with BC from the TCGA cohort (median OS, low-risk group: 26.9 months; moderate-risk group: 41.7 months; high-risk group: 22.1 months; *P*=0.55) (**Supplementary Figure 6B**). These findings demonstrated the specificity of the predictive value of the *TP53*/*PIK3CA*/*ATM* mutation classifier in patients with BC responding to ICI therapy.

### Gene Signatures and Pathway Enrichment Analysis by the *TP53*/*PIK3CA*/*ATM* Mutation Classifier

**Figure 5:**
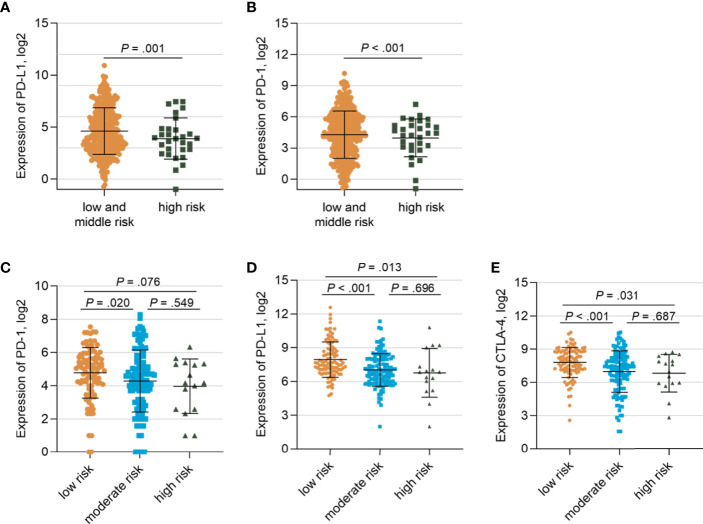
Gene Expressions of Patients in the TCGA Cohort and IMvigor210 cohort stratified by the *TP53/PIK3CA/ATM* Mutation Classifier **(A)** Comparison of the expression of PD-1 between the low- and moderate-risk group and the high-risk group. **(B)** Comparison of the expression of PD-L1 between the low and moderate -risk group and the high-risk group. **(C)** Comparison of the expression of PD-1 among three risk groups in IMvigor210 cohort. **(D)** Comparison of the expression of PD-L1 among three risk groups in IMvigor210 cohort. **(E)** Comparison of the expression of CTLA-4 among three risk groups in IMvigor210 cohort.

To further investigate the gene signatures of the *TP53*/*PIK3CA*/*ATM* mutation classifier based on RNA-seq data, we utilized the TCGA cohort and IMvigor210 cohort. Lower expression of PD-1 (FPKM: 27.7 *vs.* 57.6, respectively; *P<*0.001) and PD-L1 (FPKM: 35.0 *vs.* 79.8, respectively; *P=*0.001) were observed in the high-risk group than the other two groups (**Figures 5A, B**) in the TCGA cohort. Similar results were found in the IMvigor210 cohort (**Figures 5C–E**). The GSEA was performed between the low-risk group and high-risk group in the TCGA cohort to appraise the hallmark gene sets, C2: KEGG gene sets and C7: immunologic signature gene sets (**Supplementary Figure 7**). Intriguingly, we found that cell cycle-related pathways, such as the E2F targets (FDR=0.06) and G2M checkpoint (FDR=0.11), were enriched in hallmark gene sets. Altered pathways from the C2 subset in the low-risk group, including homologous recombination (FDR=0.03), pyrimidine metabolism (FDR=0.06), purine metabolism (FDR=0.08), and DNA replication (FDR=0.07), were related to the genomic stability status. Variation in these pathways may lead to higher TMB and MSI. In the C7 immunologic sets, 114 gene sets were significantly enriched in the low-risk group (FDR<0.25), indicating an intense immune activation status. Correspondingly, there was no set found in the high-risk group. In general, the GSEA uncovered enriched pathways in cell cycle, DNA repair, and immune infiltration.

### Immune Infiltration Analysis by the *TP53*/*PIK3CA*/*ATM* Mutation Classifier

**Figure 6:**
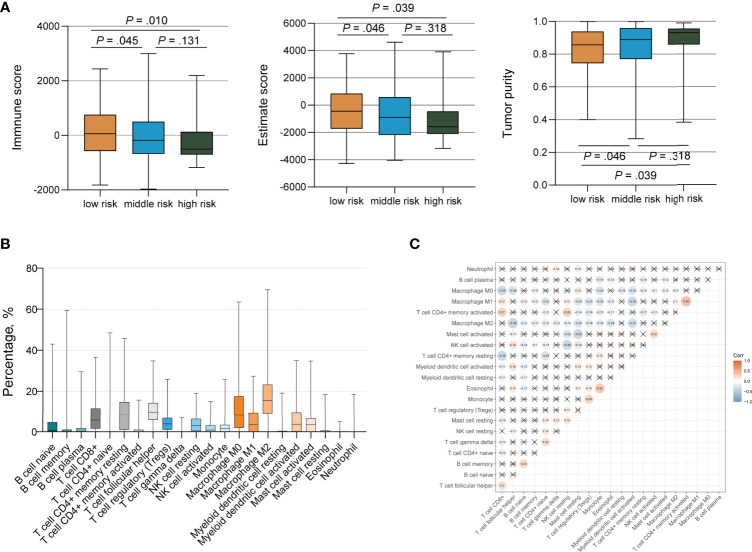
Immune Infiltration of Tumor Cells by the *TP53/PIK3CA/ATM* Mutation Classifier. **(A)** Immune scores, estimate scores, and tumor purity distribution by the ESTIMATE algorithm. **(B)** Proportion of 22 immune cell subsets by the CIBERSORT algorithm. **(C)** Correlation heatmap of 22 immune cell subsets by the CIBERSORT algorithm. The blue color represents negative correlation, while the red color represents positive correlation. Correlations with a *P* value ≥ 0.05 were marked with a cross.

**Figure 7:**
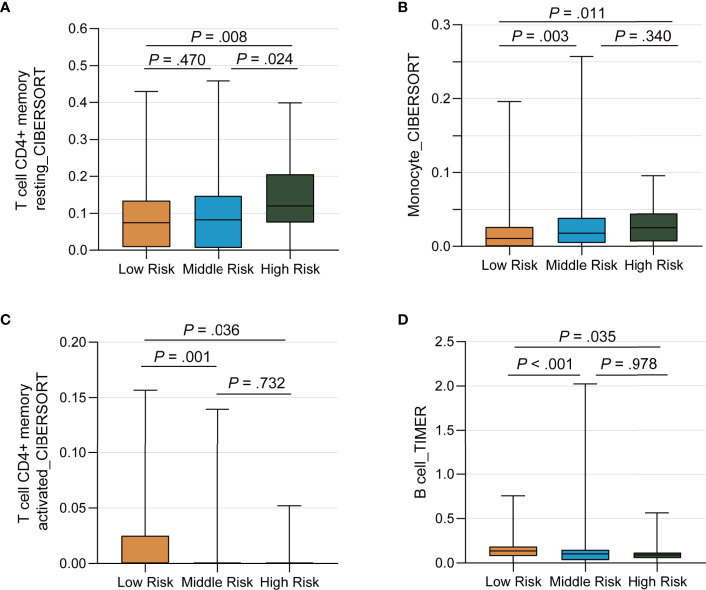
Differential Distribution of Immune Infiltration Cells by the *TP53/PIK3CA/ATM* Mutation Classifier. **(A–C)** The fraction of resting memory CD4+ T cells, activated memory CD4+ T cells, and monocytes by the CIBERSORT algorithm, respectively. **(D)** The fraction of B cells by the TIMER algorithm.

We ran the ESTIMATE algorithm to predict the tumor purity and infer the fractions of stromal cells and immune cells in the TCGA cohort and IMvigor210 cohort. There were conspicuous statistical differences in immune scores, estimate scores, and tumor purity among three risk groups in the TCGA cohort (**Figure 6A**). The low-risk group was infiltrated by more immune cells and consisted of fewer tumor cells than the high-risk group, in accordance with the better response to ICI therapy. Among the 22 immune cell subsets of the CIBERSORT algorithm in the TCGA cohort, M2 macrophages, M0 macrophages, helper T cells, resting memory CD4+ T cells, and CD8+ T cells were the five most abundant immune cell components, which accounted for >57.4% of immune cells (**Figure 6B**). The correlation between different fractions of immune cell subsets ranged from −0.38 to 0.38 (**Figure 6C**). The proportion of 22 immune cell subsets by the CIBERSORT algorithm and immune correlation heatmap in the IMvigor210 cohort were shown in **Supplementary Figures 8A, B**. The fractions of resting memory CD4+ T cells and monocytes were higher in the high-risk group of the TCGA cohort (**Figures 7A, B**). In contrast, the fraction of activated memory CD4+ T cells was higher in the low-risk group (**Figure 7C**). Higher expression of activated NK cells, M1 Macrophages, and gamma delta T cells were also observed in low-risk group of the IMvigor210 cohort (**Supplementary Figures 8C–E**). In addition, we utilized the TIMER algorithm and noticed a higher B cell fraction in the low-risk group of the TCGA cohort and a higher neutrophil fraction in the low-risk group of IMvigor210 cohort (**Figure 7D**; **Supplementary Figure 8F**). The heatmap of immune cells by the TIMER and CIBERSORT algorithms in the TCGA cohort also revealed more immune infiltration in the low-risk group (**Supplementary Figure 9**). Importantly, the low-risk group showed a more active immune response status compared with the high-risk group in both two cohorts.

## Discussion

Over the past few decades, platinum‐based chemotherapy has become the standard option for the systemic management of muscle‐invasive and advanced BC (29). However, with the rapid development in genomic sequencing technology in recent years, ICI therapy has shown great potential in advanced BC patients with high TMB. A subset of patients could benefit from durable response to ICI therapy. Then overcoming innate and adaptive resistance to therapy has been a top priority for investigators. Therefore, the discovery of novel biomarkers for predicting the therapeutic response to ICI therapy is urgently warranted. Gene expression signatures, MSI and TMB, have been proven to be effective (6, 8, 30, 31). Other indicators under evaluation include tumor-infiltrated lymphocytes, neoantigen burden, and gastrointestinal microbiome (32–35). In this study, we identified the top 20 frequently mutated genes in five BC cohorts, and established a *TP53*/*PIK3CA*/*ATM* mutation classifier according to the MSKCC ICI cohort. We subsequently confirmed the efficacy of the classifier in the validation set, and investigated its molecular profile and immune infiltration in the TCGA cohort and IMvigor 210 cohort. It could be concluded that BC patients with lower risk scores had a longer survival. Importantly, it seemed to only work on BC patients treated with ICIs. Moreover, BC patients with high-risk scores appeared to have a poorer immune infiltration than those with low- or moderate-risk scores.

Genome instability and mutation is a brand-new hallmark of cancers (36, 37). According to a study of the mutational landscape across 12 major types of cancer from TCGA program, *TP53* (41%) and *PIK3CA* (20%) are the top two most commonly mutated genes (38). The inclusion of these two genes improves the universality of the classifier. M. Choi et al. found that *PIK3CA* mutations were related to remarkably reduced peritumoral PD-1 and tumoral *PD-L1* in lung squamous cell carcinoma (LUSC) (39). Tao et al. also reported that *PIK3CA* mutations were connected with significantly lower infiltration of macrophage in LUSC (40). Generally speaking, *PIK3CA* mutations led to the activation of PI3K-AKT-mTOR signaling pathway. Activated PI3K-AKT-mTOR pathway increased production of free fatty acids, which were more effectively consumed by regulatory T cells and decreased effector T cell infiltration (41). Moreover, Borcoman et al. reported that PI3K inhibitors could increase the immune infiltration of BC with *PIK3CA* mutations, thus restoring the sensitivity to ICIs (42). These findings may partially interpret the attenuated response to ICI therapy in patients with *PIK3CA* mutations. *ATM* plays an important role in the regulation of the DNA damage response mechanism. Mutations in this gene are related to ataxia telangiectasia and tumor response to treatment, such as BC, prostate cancer, etc. (43). Its crucial role in response to double-strand breaks (DSB) has been noted, followed by the phosphorylation of extensive downstream signaling pathways (44). Phosphorylated p53 is subsequently stabilized and accumulated in the nucleus, acting as a central transcription factor for the regulation of DNA repair (45). The repair of DSB mainly involves homologous recombination and non-homologous end joining (46). Homologous recombination deficiency caused by mutation of *ATM*/*TP53* results in a preference to non-homologous end joining, which is a type of less accurate DSB repair and associated with higher TMB. Yu et al. discovered that comutation of *TP53* and *ATM* was associated with increased responses to ICIs in non–small cell lung cancer (9), while comutation of *TP53* and *ATM* was also divided into low-risk group in our classifier.

In our study, the low-risk group yielded a higher immune score than the other two groups, according to the ESTIMATE algorithm. Immune infiltration analysis revealed higher expression of B cells and activated memory CD4+ T cells, as well as lower expression of resting memory CD4+ T cells and monocytes in the low-risk group. Recent studies have shown that overexpression of monocytes enhanced the levels of glycolysis in the peritumoral area, leading to impaired cytotoxic T lymphocyte responses in tumors (47). Excessive production of interleukin-10 by monocytes leads to reversible dysfunction of CD4+ T cells (48). Accordingly, correlation analysis showed a positive correlation between monocytes and regulatory T cells. Importantly, the low-risk group exhibited a relatively more activated immune status than the high-risk group in two cohorts, indicating a greater benefit from ICI therapy.

The GSEA showed enriched pathways in cell cycle, DNA repair, and tumor metabolism in the low-risk group. Generally speaking, deficiencies in DNA damage response increase the tumor mutation load and the generation of neoantigen burden, which helps the immune system to recognize the tumor. The effect of *ATM*/*TP53* mutation may be amplified by the pathways, resulting in an accelerated accumulation of the mutation burden.

Compared with traditional chemotherapy and targeted therapy, ICI therapy requires a longer period of time to show its curative effects. In a retrospective study of 262 patients treated with anti-*PD-L1* monotherapy, 48 of 76 responder patients presented an objective response at 3 months (49). Our approach can distinguish patients who are sensitive to ICI therapy and accelerate the benefits of treatment. Meanwhile, we need to pay closer attention to the management of patients in the high-risk group. Since an undesirable curative response to ICI therapy is foreseeable, traditional chemotherapy or combined treatment with chemotherapy and targeted therapy need to be taken into consideration at the early stage of management.

Despite these promising findings, the present study had several limitations. Firstly, we conducted a retrospective study based on online published data. The findings should be verified in prospective studies with larger cohorts in the future. Secondly, we conducted substantial immune infiltration analyses through bioinformatics algorithms like the ESTIMATE algorithm, the CIBERSORT algorithm and the TIMER algorithm. The fidelity of reference profiles is the limitation of the signature gene-based algorithms. The results may deviate in tumor-induced dysregulation, phenotypic plasticity, and tumor heterogeneity. Besides, we could not ignore the different results among algorithms. Modified algorithms are needed to reconcile differences among the existing algorithms. Thirdly, the mechanisms underlying the *TP53*/*PIK3CA*/*ATM* mutations and response to ICI therapy remain unclear. Further investigation is warranted to understand the full impact of *TP53*/*PIK3CA*/*ATM* mutations.

In summary, our *TP53*/*PIK3CA*/*ATM* mutation classifier could predict the therapeutic response of patients with BC to ICI therapy. Only BC patients treated with ICIs and with lower risk scores had a longer survival. Stronger immune infiltration was observed in the low-risk group of the classifier. The present findings have important implications for clinical treatment strategy.

## Conclusion

We established a *TP53/PIK3CA/ATM* mutation classifier to predict the therapeutic response of patients with BC to ICI therapy. This classifier has the potential to become a useful complement to TMB and guide the clinical treatment decision according to the levels of risk.

## Data Availability Statement

The original contributions presented in the study are included in the article/**Supplementary Material**. Further inquiries can be directed to the corresponding authors.

## Ethics Statement

Ethical review and approval was not required for the study on human participants in accordance with the local legislation and institutional requirements. Written informed consent for participation was not required for this study in accordance with the national legislation and the institutional requirements.

## Author Contributions

J-HL and Y-HP made contributions to conception, design, statistical analysis and drafting of the manuscript. J-XZ, XC, and FL acquired the data. J-ZC, WC, and YC provide administrative, technical support. YC, WC, and J-HL supervised the study. All authors contributed to the article and approved the submitted version.

## Funding

The article was supported by the National Natural Science Foundation of China (81725016, 81872094, 81772718, 81572522); Natural Science Foundation of Guangdong Province (2017B020227004). The funders had no role in the design and conduct of the study; collection, management, analysis, and interpretation of the data; preparation, review, or approval of the manuscript; and decision to submit the manuscript for publication.

## Conflict of Interest

The authors declare that the research was conducted in the absence of any commercial or financial relationships that could be construed as a potential conflict of interest.

## Publisher’s Note

All claims expressed in this article are solely those of the authors and do not necessarily represent those of their affiliated organizations, or those of the publisher, the editors and the reviewers. Any product that may be evaluated in this article, or claim that may be made by its manufacturer, is not guaranteed or endorsed by the publisher.

## Footnotes

1
http://www.cbioportal.org/study/summary?id=tmb_mskcc_2018

2
http://www.cbioportal.org/study/summary?id=msk_impact_2017

3
https://portal.gdc.cancer.gov

4
http://www.cbioportal.org/study/summary?id=blca_tcga_pub_2017

5
http://www.cbioportal.org/study/summary?id=blca_cornell_2016

6
http://www.cbioportal.org/study/summary?id=blca_dfarber_mskcc_2014

7
http://www.cbioportal.org/study/summary?id=mixed_allen_2018

8
http://research-pub.gene.com/IMvigor210CoreBiologies

9
https://string-db.org

10
http://cibersort.stanford.edu

11
http://timer.cistrome.org
