# Supplementary material for: Predictive Value of the TP53/PIK3CA/ATM Mutation Classifier for Patients With Bladder Cancer Responding to Immune Checkpoint Inhibitor Therapy
Source: Front Immunol. 2021 Aug 4;12:643282. doi: 10.3389/fimmu.2021.643282 (PMC8371040; doi:10.3389/fimmu.2021.643282)
Supplement: Supplementary file 1 [file DataSheet_1.docx]

Supplementary Material

**Supplementary Figure 1.** Data Sources

**Supplementary Figure 2.** Mutation Pattern and Frequencies in Patients with Bladder Cancer from Five Cohorts (n=881)

**Supplementary Figure 3.** PPI Network of the Top 20 Frequently Mutated Genes in Bladder Cancer

**Supplementary Figure 4.** The Optimal Threshold of Risk Score for Overall Survival in the MSKCC ICI Cohort Determined by the X-tile Model

**Supplementary Figure 5.** Correlation between the Risk Score and TMB in the MSKCC ICI Cohort

**Supplementary Figure 6.** Overall Survival of Patients with Bladder Cancer by the *TP53/PIK3CA/ATM* Mutation Classifier in Non-ICI Cohorts

**Supplementary Figure 7.** Gene Set Enrichment Analysis of Patients with Bladder Cancer in the MSKCC ICI Cohort by the *TP53/PIK3CA/ATM* Mutation Classifier

**Supplementary Figure 8.** Immune Infiltration of Tumor Cells by the *TP53/PIK3CA/ATM* Mutation Classifier in the IMvigor210 Cohort

**Supplementary Figure 9.** Heatmap of Immune Cells by the TIMER and CIBERSORT Algorithms in the TCGA Cohort

**Supplementary Table 1.** Univariate Analysis of the Top 20 Frequently Mutated Genes in Patients with Bladder Urothelial Carcinoma from the MSKCC ICI Cohort (n=215)

**Supplementary Table 2.** Multivariate Cox Regression Analyses of Four Potential Prognostic Factors in Patients with Bladder Cancer from the MSKCC ICI Cohort (n=215)

**Supplementary Table 3.** Multivariate Cox Regression Analyses of Three Prognostic Candidates in Patients with Bladder Cancer from the MSKCC ICI Cohort (n=215)

**Supplementary Table 4.** Multivariate Cox Regression Analyses of Patients with Bladder Cancer in the MSKCC ICI Therapy Cohort (n=215)

**Supplementary Table 5.** Univariate and Multivariate Cox Regression Analyses of TMB with OS in the MSKCC ICI Cohort (n=215)

**Supplementary Table 6.** Univariate and Multivariate Cox Regression Analyses of Patients with Bladder Cancer in the Validation Set (n=263)

**Supplementary Figure 1.** Data Sources

**
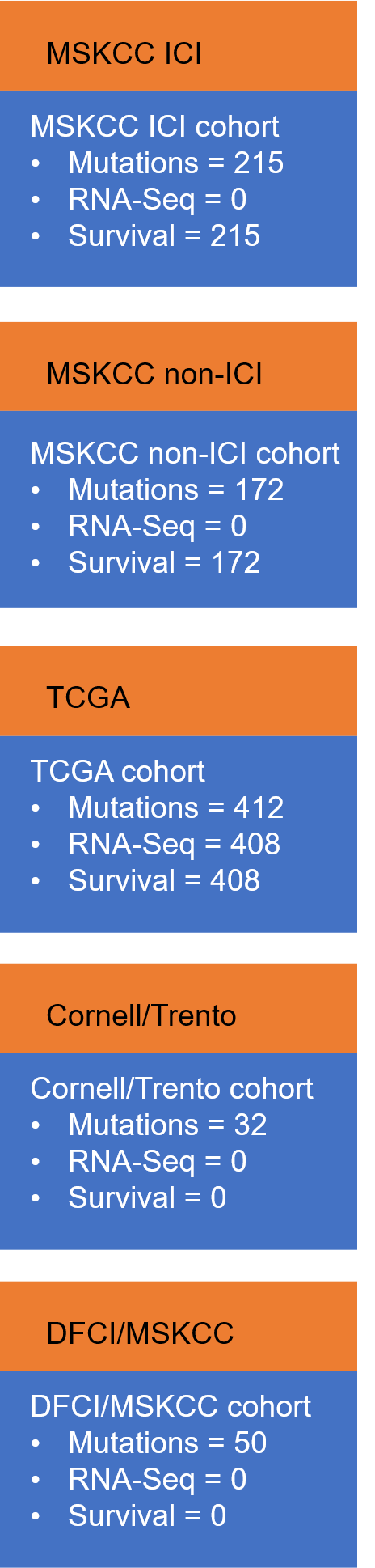
**

**Supplementary Figure 2.** Mutation Pattern and Frequencies in Patients with Bladder Cancer from Five Cohorts

**
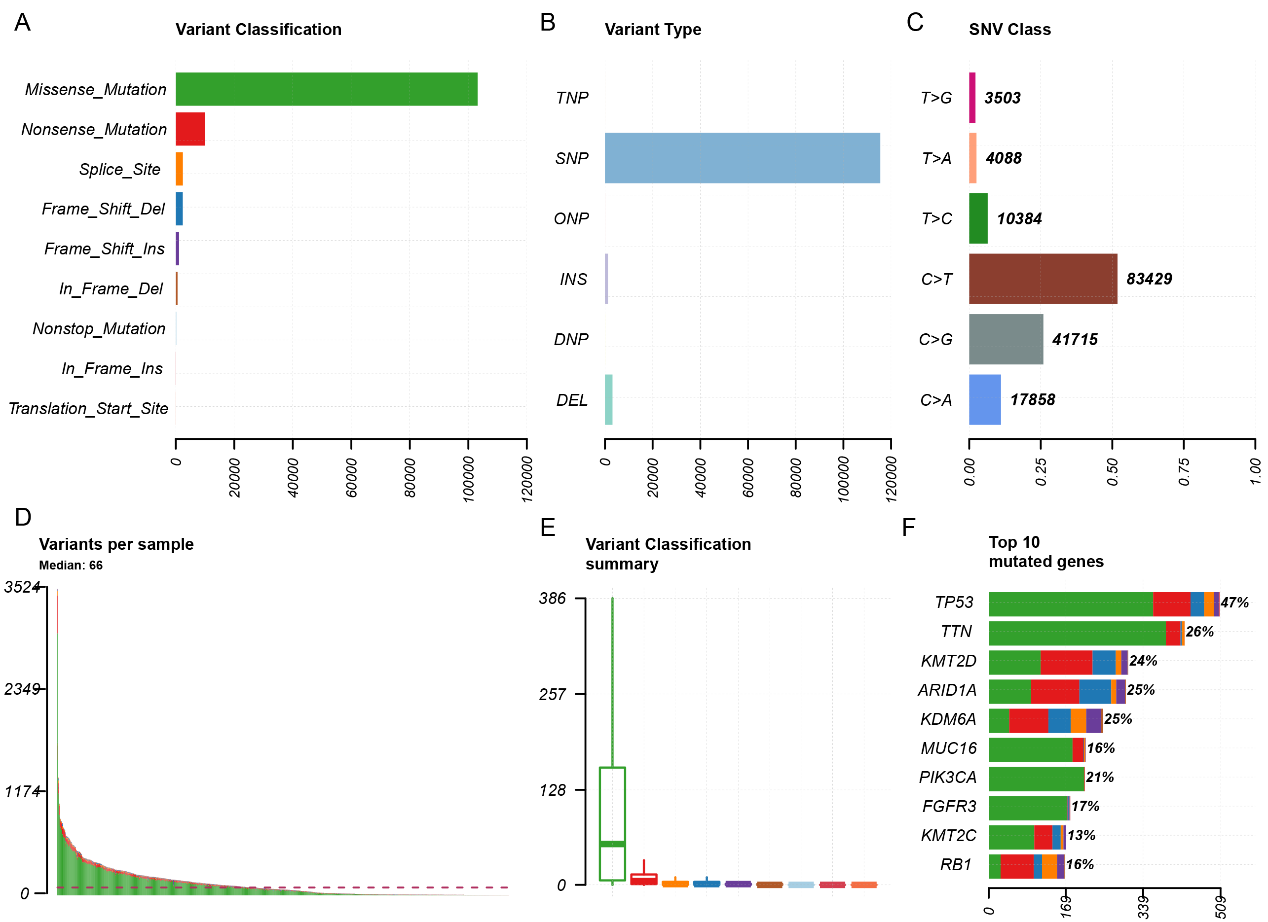
**

**Supplementary Figure 3.** PPI Network of the top 20 Frequently Mutated Genes in Bladder Cancer

The green color represents more edgecounts, while the yellow color represents less edgecounts.

**
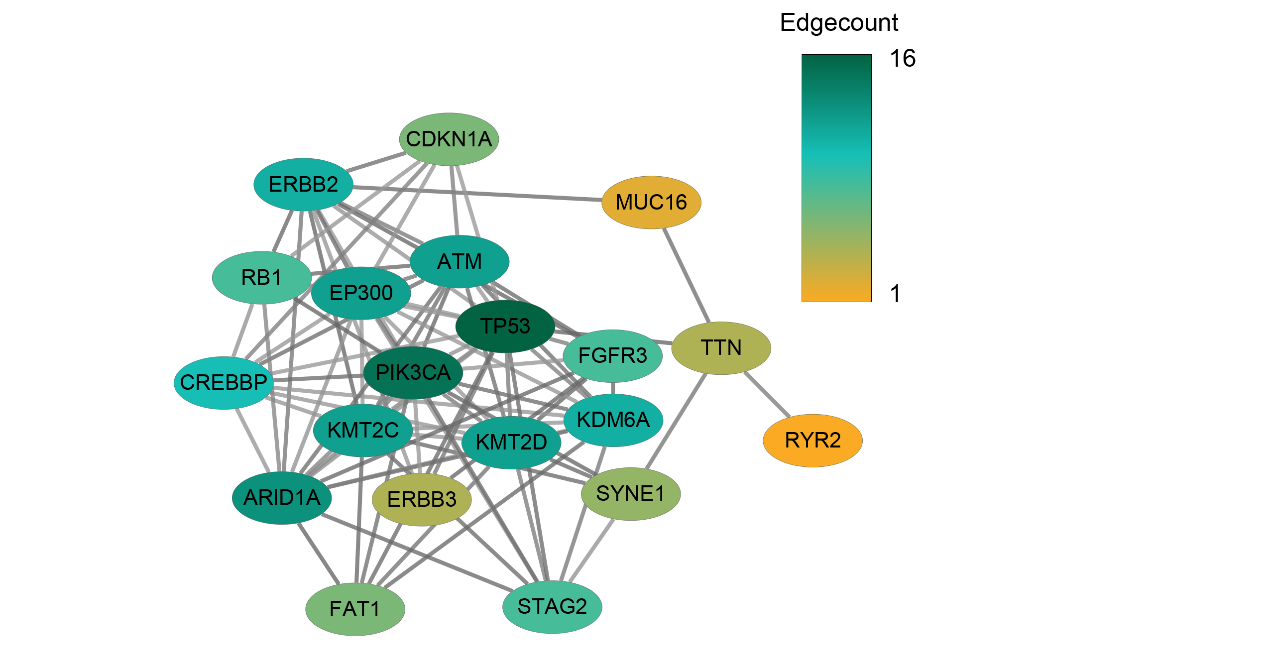
**

**Supplementary Figure 4.** The Optimal Threshold of Risk Scores for Overall Survival in the MSKCC ICI Cohort Determined by the X-tile Model

**(A)** X-tile plot based on risk scores for overall survival. **(B)** The histogram showed the optimal cut-off points, 0 and 0.07. **(C)** Kaplan-Meier curve based on the optimal division. **(D)** Statistics generated by X-tile software.

**
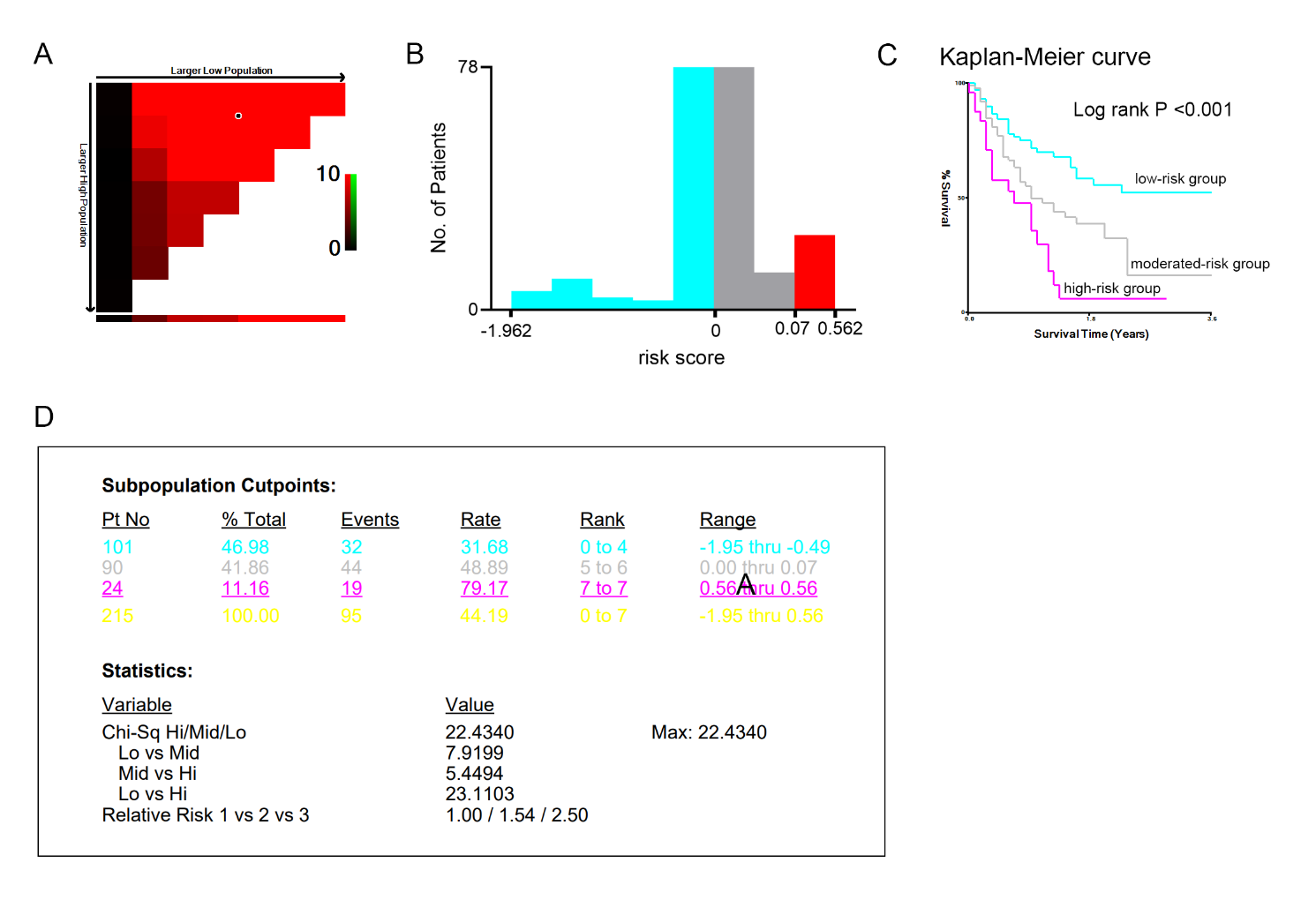
**

**Supplementary Figure 5.** Correlation between the Risk Score and TMB in the MSKCC ICI Cohort

**
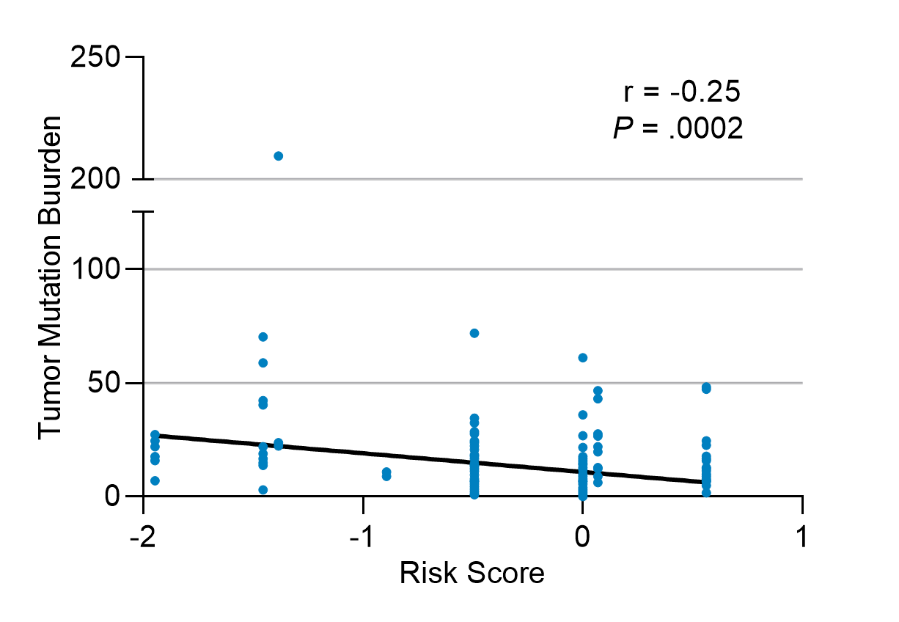
**

**Supplementary Figure 6.** Overall Survival of Patients with Bladder Cancer by the *TP53/PIK3CA/ATM* Mutation Classifier in Non-ICI Cohorts

**(A)** Kaplan–Meier curves of overall survival in patients with bladder cancer from the MSKCC non-ICI therapy cohort (n=172). The median overall survival was 30.5 months (95% CI: 17.8–43.1 months) in the moderate-risk group and not reached in the other two groups. **(B)** Kaplan–Meier curves of overall survival in patients with bladder cancer from TCGA cohort (n=408). The median overall survival was 22.1 months (95% CI: 0.0–61.1 months) in the high-risk group, 41.7 months (95% CI: 29.8–53.6 months) in the moderate-risk group, and 26.9 months (95% CI: 3.8–50.1 months) in the low-risk group.

B

A

TCGA cohort

MSKCC non-ICI therapy cohort

**Supplementary Figure 7.** Gene Set Enrichment Analysis of Patients with Bladder Cancer in the TCGA Cohort by the *TP53/PIK3CA/ATM* Mutation Classifier**
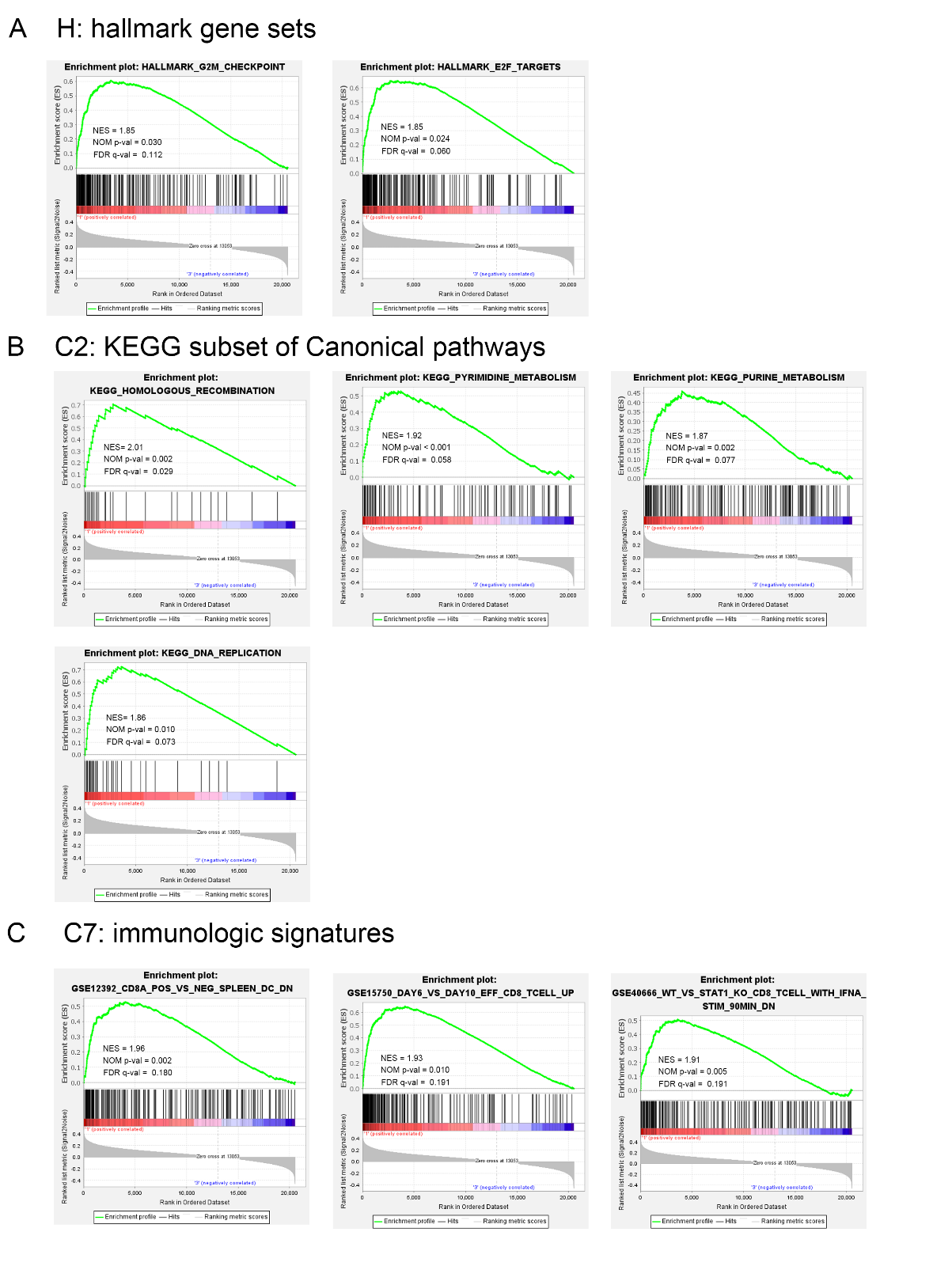
**


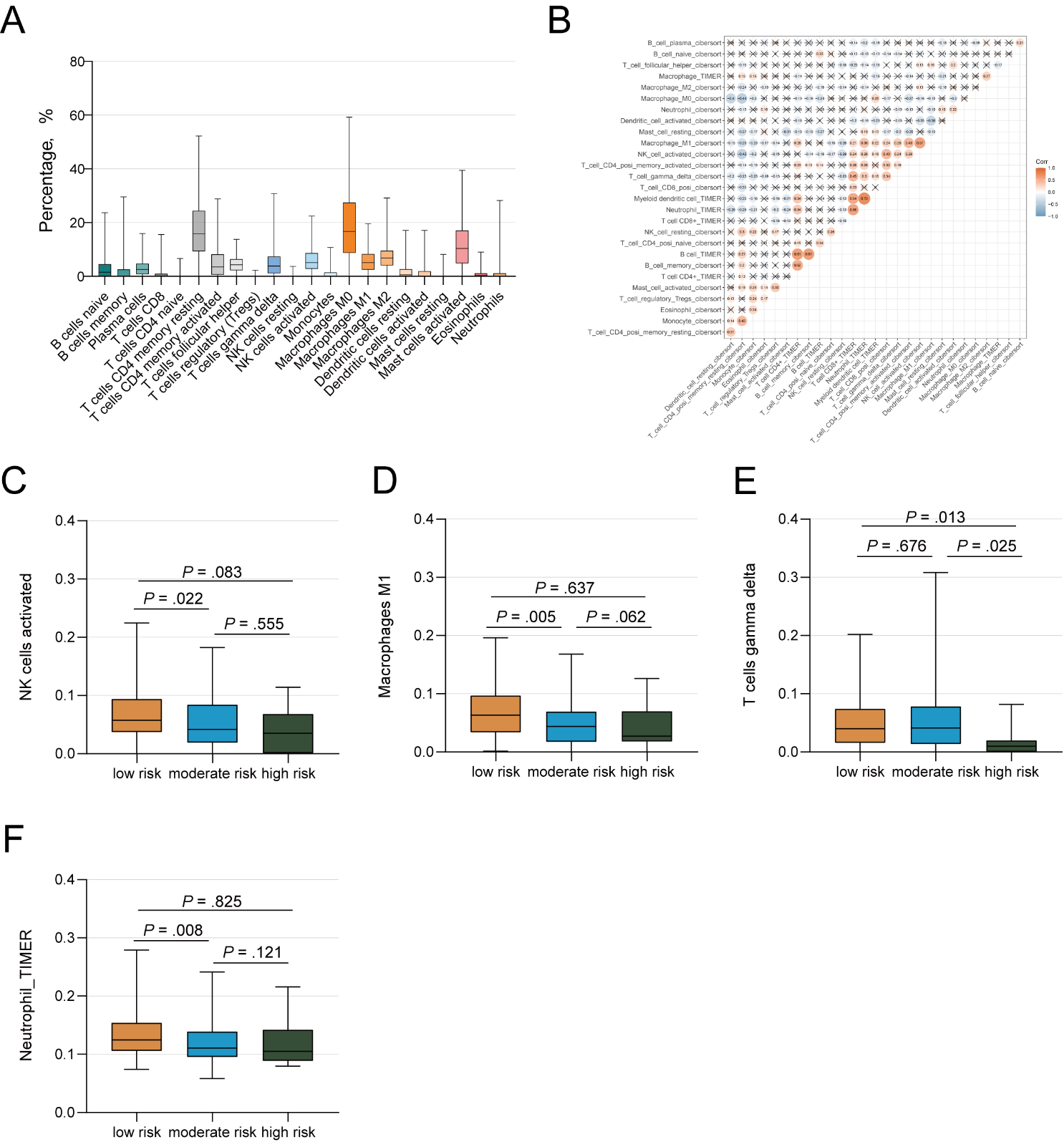
**Supplementary Figure 8.** Immune Infiltration of Tumor Cells by the *TP53/PIK3CA/ATM* Mutation Classifier in the IMvigor210 Cohort **(A)** Proportion of 22 immune cell subsets by the CIBERSORT algorithm. **(B)** Correlation heatmap of 22 immune cell subsets by the CIBERSORT algorithm. The blue color represents negative correlation, while the red color represents positive correlation. Correlations with a *P* value ≥ 0.05 were marked with a cross. **(C-E)** The fraction of activated NK cells, M1 Macrophages and gamma delta T cells by the CIBERSORT algorithm, respectively. **(F)** The fraction of Neutrophil by the TIMER algorithm.

**Supplementary Figure 9.** Heatmap of Immune Cells in the TCGA cohort by the TIMER and CIBERSORT Algorithms in the TCGA Cohort

**
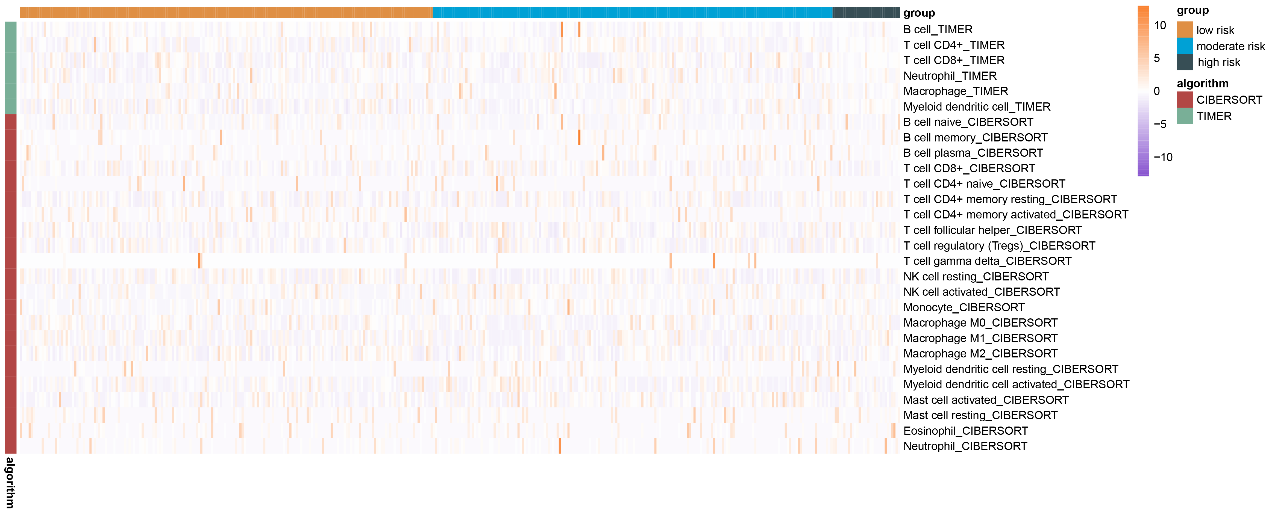
**

**Supplementary Table 1.** Univariate Analysis of the Top 20 Frequently Mutated Genes in Patients with Bladder Urothelial Carcinoma from the MSKCC ICI Cohort (n=215)

| **Gene Name** | ***P* Value** | **HR** |  | **95% CI for HR** | |  |
| --- | --- | --- | --- | --- | --- | --- |
|  |  |  |  | **Lower** | **Upper** |  |
| *TP53* | 0.031 | 0.633 |  | 0.418 | 0.960 |  |
| *TTN* | - | - |  | - | - |  |
| *KDM6A* | 0.230 | 0.735 |  | 0.444 | 1.216 |  |
| *ARID1A* | 0.592 | 0.881 |  | 0.554 | 1.401 |  |
| *KMT2D* | 0.491 | 0.856 |  | 0.551 | 1.332 |  |
| *PIK3CA* | 0.033 | 1.635 |  | 1.041 | 2.568 |  |
| *FGFR3* | 0.546 | 1.161 |  | 0.715 | 1.887 |  |
| *MUC16* | - | - |  | - | - |  |
| *RB1* | 0.696 | 0.882 |  | 0.470 | 1.654 |  |
| *EP300* | 0.185 | 0.594 |  | 0.275 | 1.284 |  |
| *KMT2C* | 0.825 | 0.938 |  | 0.532 | 1.655 |  |
| *CREBBP* | 0.020 | 0.421 |  | 0.204 | 0.871 |  |
| *ATM* | 0.012 | 0.277 |  | 0.101 | 0.754 |  |
| *ERBB2* | 0.696 | 1.134 |  | 0.604 | 2.126 |  |
| *ERBB3* | 0.083 | 0.481 |  | 0.210 | 1.101 |  |
| *STAG2* | 0.189 | 0.547 |  | 0.222 | 1.347 |  |
| *CDKN1A* | 0.641 | 0.861 |  | 0.459 | 1.616 |  |
| *FAT1* | 0.067 | 0.460 |  | 0.201 | 1.055 |  |
| *SYNE1* | - | - |  | - | - |  |
| *RYR2* | - | - |  | - | - |  |
| HR=hazard ratio. CI= confidence interval | | | | | | |

**Supplementary Table 2.** Multivariate Cox Regression Analyses of Four Potential Prognostic Factors in Patients with Bladder Cancer from the MSKCC ICI Cohort (n=215)

| **Variables** | ***P* Value** | **HR** | **95.0% CI for HR** | |
| --- | --- | --- | --- | --- |
|  |  |  | **Lower** | **Upper** |
| *TP53* | 0.034 | 0.634 | 0.416 | 0.967 |
| *PIK3CA* | 0.013 | 1.782 | 1.129 | 2.813 |
| *CREBBP* | 0.124 | 0.558 | 0.265 | 1.174 |
| *ATM* | 0.016 | 0.282 | 0.101 | 0.787 |
| HR=hazard ratio. CI= confidence interval. | | | | |

**Supplementary Table 3.** Multivariate Cox Regression Analyses of Three Prognostic Candidates in Patients with Bladder Cancer from the MSKCC ICI Cohort (n=215)

| **Variables** | | **Coefficient** | **SE** | ***P* Value** | **HR** | **95.0% CI for HR** | |
| --- | --- | --- | --- | --- | --- | --- | --- |
|  |  |  |  |  |  | **Lower** | **Upper** |
|  | *TP53* | -0.492 | 0.215 | 0.022 | 0.611 | 0.401 | 0.931 |
|  | *PIK3CA* | 0.562 | 0.232 | 0.015 | 1.755 | 1.113 | 2.766 |
|  | *ATM* | -1.454 | 0.514 | 0.005 | 0.234 | 0.085 | 0.639 |
| HR=hazard ratio. CI= confidence interval. SE= standard errors. | | | | | | | |

**Supplementary Table 4.** Multivariate Cox Regression Analyses of Patients with Bladder Cancer in the MSKCC ICI Therapy Cohort (n=215)

| **Parameters** | **Multivariate analysis** | |
| --- | --- | --- |
|  | **HR (95%CI)** | **P Value** |
| Sex (men vs women) | 0·95 (0·58-1.55) | 0.555 |
| Age (≥60 years vs <60 years) | 1.23(0.76-1.97) | 0.398 |
| Treatment (PD-1/PD-L1 vs combo) | 0.82(0.45-1.49) | 0.507 |
| Risk Score (low risk vs moderate risk vs high risk) | 1.78(1.35-2.36) | <0·0001 |
| TMB (higher vs lower subgroup) | 0.60(0.35-1.01) | 0.054 |
| HR=hazard ratio. CI= confidence interval | | |

**Supplementary Table 5.** Univariate and Multivariate Cox Regression Analyses of TMB with OS in the MSKCC ICI Cohort (n=215)

| **Parameters** | **TMB-low status (n=191)** | | | | |  | **TMB-high status (n=51)** | | | | |
| --- | --- | --- | --- | --- | --- | --- | --- | --- | --- | --- | --- |
|  | **Univariate analysis** | |  | **Multivariate analysis** | |  | **Univariate analysis** | |  | **Multivariate analysis** | |
|  | **HR (95%CI)** | ***P* Value** |  | **HR (95%CI)** | ***P* Value** |  | **HR (95%CI)** | ***P* Value** |  | **HR (95%CI)** | ***P* Value** |
| Sex (men vs women) | 0.90(0.54-0.50) | 0.689 |  | 1.11(0.66-1.88) | 0.687 |  | 0.59(0.14-2.61) | 0.49 |  | 0.34(0.08-1.56) | 0.167 |
| Age  (≥60 years vs <60 years) | 1.17(0.70-1.94) | 0.553 |  | 1.22(0.73-2.03) | 0.451 |  | 1.14(0.33-3.99) | 0.837 |  | 1.40(0.39-5.03) | 0.608 |
| Treatment  (PD-1/PD-L1 vs combo) | 0.77(0.39-1.53) | 0.454 |  | 0.69(0.33-1.45) | 0.333 |  | 1.66(0.33-4.08) | 0.823 |  | 0.68(0.18-2.53) | 0.563 |
| Risk Score  (low-risk vs moderate -risk vs high-risk) | 1.65(1.22-2.23) | 0.001 |  | 1.65(1.20-2.25) | 0.002 |  | 2.43(1.30-4.54) | 0.006 |  | 2.66(1.43-4.94) | 0.002 |
| HR=hazard ratio. CI= confidence interval | | | | | | | | | | | |

**Supplementary Table 6.** Univariate and Multivariate Cox Regression Analyses of Patients with Bladder Cancer in the Validation Set (n=263)

| **Parameters** | **Univariate analysis** | |  | **Multivariate analysis** | |
| --- | --- | --- | --- | --- | --- |
|  | **HR (95%CI)** | **P Value** |  | **HR (95%CI)** | **P Value** |
| Sex (men vs women) | 0.91 (0.61-1.35) | 0.647 |  | 0.92(0.62-1.36) | 0.665 |
| Risk Score (low-risk vs moderate-risk vs high-risk) | 1.39(1.08-1.79) | 0.011 |  | 1.37(1.06-1.77) | 0.017 |
| TMB (higher vs lower subgroup) | 0.70(0.47-1.07) | 0.097 |  | 0.73(0.48-1.11) | 0.145 |
| HR=hazard ratio. CI= confidence interval | | |  |  |  |
